# Optimizing Molecular-Targeted Therapies in Ovarian Cancer: The Renewed Surge of Interest in Ovarian Cancer Biomarkers and Cell Signaling Pathways

**DOI:** 10.1155/2012/737981

**Published:** 2012-02-06

**Authors:** Donavon Hiss

**Affiliations:** Molecular Oncology Research Laboratory, Department of Medical BioSciences, University of the Western Cape, Bellville 7535, South Africa

## Abstract

The hallmarks of ovarian cancer encompass the development of resistance, disease recurrence and poor prognosis. Ovarian cancer cells express gene signatures which pose significant challenges for cancer drug development, therapeutics, prevention and management. Despite enhancements in contemporary tumor debulking surgery, tentative combination regimens and abdominal radiation which can achieve beneficial response rates, the majority of ovarian cancer patients not only experience adverse effects, but also eventually relapse. Therefore, additional therapeutic possibilities need to be explored to minimize adverse events and prolong progression-free and overall response rates in ovarian cancer patients. Currently, a revival in cancer drug discovery is devoted to identifying diagnostic and prognostic ovarian cancer biomarkers. However, the sensitivity and reliability of such biomarkers may be complicated by mutations in the *BRCA1* or *BRCA2* genes, diverse genetic risk factors, unidentified initiation and progression elements, molecular tumor heterogeneity and disease staging. There is thus a dire need to expand existing ovarian cancer therapies with broad-spectrum and individualized molecular targeted approaches. The aim of this review is to profile recent developments in our understanding of the interrelationships among selected ovarian tumor biomarkers, heterogeneous expression signatures and related molecular signal transduction pathways, and their translation into more efficacious targeted treatment rationales.

## 1. Introduction

Ovarian cancer is the major cause of gynecological cancer deaths worldwide [1–6]. It is widely accepted that the distinctive genotypic and phenotypic characteristics of ovarian cancer not only promote its metastatic potential but are also responsible for the development of resistance to conventional modes of cancer therapy, disease recurrence, and poor prognosis [2, 4, 7–19]. In particular, epithelial ovarian cancer (EOC) presents a considerable impediment to successful treatment outcome because of its propensity to embark on a program of epithelial-to-mesenchymal transition (EMT), a transdifferentiation process that is almost invariably associated with tumor progression and invasiveness [2, 15, 19–24]. 

Furthermore, self-renewing ovarian cancer stem cells (OCSCs) or ovarian cancer-initiating cells (OCICs), as well as mesenchymal stem cells (MSCs), have been implicated in ovarian tumorigenesis, intra- and extraperitoneal metastases, and chemoresistance [2, 19, 25–27]. Since cancer stem cells (CSCs) are predominantly quiescent, have upregulated DNA repair capacity, are noncommittal to apoptosis, and overexpress ATP-binding cassette (ABC) drug efflux transporters, for example, ABCG1 (MDR1/P-glycoprotein/Pgp), ABCG2, and breast cancer resistance protein (BCRP), and a profusion of cancer gene signatures, they sustain the succession of clonal tumor cell proliferation and repopulation in the tumor microenvironment [2, 22, 25, 26, 28–38]. Many CSC-derived or EMT-induced tumors, including ovarian cancer, also express this aggressive, malignant, and multidrug resistance (MDR) phenotype and other tumor prosurvival repertoires which pose significant challenges for cancer drug development, therapeutics, prevention, and management [2, 19–22, 28, 33, 34, 39].

The optimal management modality for ovarian cancer includes histopathological diagnosis and staging, debulking (surgical resection) of the tumor, and several cycles of intravenous (IV) or intraperitoneal (IP) chemotherapy with carboplatin and paclitaxel at maximum tolerated doses (MTDs), followed by maintenance or salvage treatments, in cases of disease recurrence [3, 12, 15, 40, 41]. Although refinements in tumor ablation procedures and IP combination chemotherapy with carboplatin and paclitaxel can achieve beneficial response rates, for example, median progression-free survival (PFS) range of 16 to 21 months and median overall survival (OS) range of 24 to 60 months, most patients with advanced disease ultimately relapse [15, 23, 40, 42–46]. Likewise, the majority of contemporary or tentative regimens of more than two cytotoxic drugs as well as low-dose chemosensitizing abdominal radiation have not yielded radically improved efficacy or significantly reduced adverse effects over the dual combination of carboplatin and paclitaxel, suggesting that other therapeutic avenues need to be explored to prolong PFS and OS rates in ovarian cancer patients [23, 39, 41, 47–55].

Recently, there has been a resurgence of efforts to identify ovarian cancer biomarkers for use in initial detection, staging, disease prognosis, molecular therapeutic targeting, and individualized clinical management of patients [14, 56–73]. Nonetheless, the sensitivity and reliability of ovarian cancer biomarkers may be confounded by several characteristics of the disease such as mutations in the *BRCA1* or *BRCA2* genes and their arcane absence in sporadic ovarian cancer, diverse genetic risk factors, unidentified initiation and progression elements, molecular tumor heterogeneity, and transition time between different stages of the disease. Correspondingly, the lack of a one-fit-all (i.e., highly sensitive and specific) biomarker for different histotypes of ovarian cancer—for example, EOC can be classified into four distinct histotypes: fallopian tube (serous), endometrium (endometrioid), endocervix (mucinous), or nests within the vagina (clear cell), coupled with differential overexpression of homeobox (Hox) genes—suggests that combination panels of biomarkers may offer greater diagnostic and prognostic probability [2, 12, 71, 73–75]. There is a critical need to develop broad-spectrum as well as individualized molecular-targeted therapies for ovarian cancers. Ingenious approaches are currently being applied to precisely map signal transduction pathways and target key molecular role players that direct ovarian tumor sensitivity and resistance to therapy and OS rates in patients. These include improved ultrasound and imaging technologies, molecular genetic analysis, as well as genomic, transcriptomic, and proteomic profiling of novel ovarian tumor biomarkers [2, 7, 14, 16, 56, 61, 72, 76–94]. In view of the complexities and variable response rates experienced with ovarian cancer patients clinically, the aim of this review is to outline recent developments in our understanding of the interrelationships among selected ovarian tumor biomarkers, heterogeneous expression signatures and related molecular signal transduction pathways, and their translation into futuristic as well as more efficacious targeted treatment rationales.

## 2. The Molecular Therapeutic Targeting Paradigm

The recurrence of ovarian tumors implies resistance to therapy regardless of encouraging response rates to cytoreductive surgery and combination chemotherapy, and most patients who relapse will eventually succumb to the disease [3, 15, 43, 44, 65, 95–98]. The poor prognosis in ovarian cancer patients may be broadly ascribed to distinct tumor histotypes or heterogeneity, disparate genomic expression profiles, and strikingly different molecular abnormalities [2, 12, 16–18, 39, 56, 69, 99–104]. Thus, the likelihood of ovarian cancer recurrence and resistance to therapy warrants serious alternative or complementary strategies to conventional oncologic modalities [1–4, 23, 42, 96, 105–107]. The potential for molecular-targeted therapy of ovarian cancers is increasingly being recognized and empirically validated [61, 108, 109]. Molecular therapeutic targeting is an approach that exploits specific hallmarks of cancers and the tumor microenvironment and their rationalization into clinically relevant and potent anticancer drugs with fewer side effects [1, 2, 23, 37, 39, 110–118]. Moreover, the application and exploitation of the dynamics of molecular-targeted system networks hold great promise for the design of personalized cancer therapies [119, 120]. This review provides a concise insight into recent advances in the molecular mechanisms of signal transduction pathways, the development MDR, DNA repair mechanisms, and tumor biomarkers of prognostic indicators and their therapeutic potential as translational targets in ovarian cancer.

## 3. Ovarian Cancer Biomarkers and CellSignaling Pathways

A number of reliable, complementary, or potential diagnostic and prognostic biomarkers have been reported to be overexpressed or deregulated in different types of ovarian cancer. These will be considered in Sections 3 and 4.

### 3.1. Breast Cancer 1 and 2 (BRCA1/2) Oncogenes

Ovarian cancers are associated with breast cancer 1 (*BRCA1*) and *BRCA2* oncogenes, variously inherited as germline mutations [121–124]. Wild-type *BRCA1*/*2* genes are critical for DNA repair by the homologous recombination (HR) pathway—hence their deletion causes genomic instability and predisposes affected females to familial breast and ovarian cancers [103, 104, 125–127]. Ovarian cancers with mutated *BRCA1/2* genes are particularly sensitive to agents that cause DNA double strand breaks (DSBs) and DNA interstrand cross-links, like the platinum compounds (e.g., cisplatin and carboplatin) and poly(ADP-ribose) polymerase (PARP) inhibitors (e.g., olaparib, iniparib, veliparib) [128–132]. It is conceivable, therefore, that secondary or reversion mutations of the *BRCA1/2* genes, through multiple complex mechanisms, may favor DNA repair by HR and increase tumor cell survival and so trigger resistance to these compounds [133–138]. 

In addition, upregulation of *ABCB1* genes encoding the P-glycoprotein drug efflux pump has been found to be responsible for acquired resistance in a genetically engineered mouse model (GEMM) for BRCA1-associated breast cancer, following prolonged exposure to olaparib [139]. Such resistance mechanisms need to be demarcated in order to realize the full potential of molecular targeting of *BRCA1*/*2* mutations in ovarian cancer [140, 141]. Nonetheless, a recent phase II clinical trial with orally active olaparib in women with confirmed genetic *BRCA1*/*2* mutations and recurrent measurable ovarian cancer has provided tangible proof of concept of the efficacy and tolerability of molecularly targeted treatment with PARP inhibitors, and validated *BRCA1/2* mutations as biomarkers for predicting responses of ovarian cancer patients to PARP inhibition [142]. Several other reports have, in the context of BRCA1^−/−^ ovarian cancers and their sensitivity to small molecule PARP inhibitors, presented preclinical and clinical evidence that the concept of synthetic lethality which defines a condition whereby two mutations, each with viable phenotypes, produce a lethal phenotype when they are combined can thus be exploited as a molecular-targeted strategy [133, 135, 143–147].

### 3.2. Vascular Endothelial Growth Factor (VEGF) and Its Receptor (VEGFR)

Tumor neovascularization or angiogenesis, a process dictated by complex cellular pathways that fine-tune proangiogenic and antiangiogenic factors (i.e., an angiogenic switch) in the tumor microenvironment, allows cancers to develop new blood vessels for nutrient and oxygen supply, elimination of metabolic waste products, growth, acquisition of an invasive phenotype, and metastastic spread [148–153]. Vascular endothelial growth factor (VEGF) and its receptor (VEGFR) occupy a position of prominence in angiogenesis signaling in normal ovarian physiology and in ovarian cancer progression [1, 2, 4, 15, 23, 65, 114, 154–156]. Therefore, inhibition of the angiogenesis signal transduction pathway via its ligands and receptors in ovarian cancers represents a perfectly cogent molecular targeting strategy [1, 4, 95, 98, 113, 153, 154, 157]. VEGF has long been recognized as a biomarker for predicting ovarian cancer patient responses to VEGF and other therapies and may as well have applications in formulating individualized therapies [4, 71, 72, 158–161]. Inhibitors of the VEGF pathway include bevacizumab (a humanized antibody that targets the ligand VEGF) and VEGF-trap (aflibercept, a high-affinity VEGFR decoy fusion protein that binds and inactivates VEGF and other ligands) [1, 3, 51, 95, 98, 114, 162, 163].

Besides blocking the VEGF pathway with VEGF antibodies, the angiogenic pathway can be targeted with small molecule VEGFR tyrosine kinase inhibitors (TKIs)—those currently used in ovarian cancer include, sorafenib, sunitinib cediranib, vandetanib, and intedanib (BIBF 1120) [7, 15, 65, 153, 154, 162, 164–166]. Since multiple ligands and their receptors are involved in neovascularization, including platelet-derived growth factor (PDGF/R), epidermal growth factor (EGFR/R), placenta growth factor (PlGF/R), KIT, fibroblast growth factor (FGF/R), and hepatocyte growth factor (HGF/R), resistance to single antiangiogenic drugs may occur in ovarian cancer patients, blocking such alternative pathways with rational drug combinations that have cross-specificity would be an appropriate molecular targeting strategy [1, 4, 15, 23, 114, 148, 150, 153, 156, 167–170].

### 3.3. The EGFR/ErbB Family of Receptor Tyrosine Kinases

In humans, the epidermal growth factor receptor EGFR/ErbB family of receptor tyrosine kinases (RTKs) comprises four members: EGFR/ErbB1/HER-1, ErbB2/Neu/HER-2, ErbB3/HER-3, and ErbB4/HER-4 [171, 172]. ErbB2 lacks ligand-binding capacity because its ectodomain is fixed and in an unfolded conformation, but it is the preferred ally for heterodimerization with EGFR to increase the duration and intensity of the signal triggered by high-affinity ligand binding to EGFR. Thus, ErbB2 is an amplifier of the ErbB signaling network [171]. Aberrant coexpression and collaboration of EGFR and ErbB2 is widespread in cancers and has been associated with poor prognosis [172–175]. Therefore, EGFR is deemed to be a useful biomarker for ovarian cancers [1, 2, 4, 61, 176, 177]. In ovarian cancers, mutant or isoforms of EGFR RTKs transactivate signaling transduction cascades such as PI3K/AKT and Ras/Raf/MEK/MAPK/ERK that result in diverse effects, including cell proliferation, dedifferentiation, adhesion, migration, invasion, angiogenesis, and apoptosis evasion [177–183]. Accepted tenets of molecular targeting of EGFR signaling in ovarian and non-ovarian cancers encompass small molecule TKIs (e.g., erlotinib, gefitinib), ATP-binding site inhibitors (e.g., CI-1033), anti-EGFR/ErbB2monoclonal antibodies (e.g., matuzumab, pertuzumab, cetuximab, trastuzumab), and multikinase inhibitors (e.g., vandetanib, sorafenib) [164, 166, 174, 184–194]. 

A recent phase II trial in women with predominantly platinum-resistant recurrent ovarian cancer concluded that vandetanib, a multikinase inhibitor designed to perturb both angiogenesis (i.e., VEGFR) and tumor cell growth (i.e., EGFR), did not produce translational clinical benefit since the drug inhibited EGFR and AKT levels in tumor biopsies, but had no effect on VEGFR [164]. Likewise, EGFR gene mutations and EGFR protein expression do not necessarily correlate with clinical outcome [182, 195–197]. Previous phase II clinical studies with imatinib and gefitinib in patients with refractory or recurrent EOC suggested that although these agents have marginal benefits as monotherapies in EOC, their ability to modulate molecular targets (e.g., EGFR, c-Kit, PDGFR, ERK, AKT) and demonstrate proof of concept corroborates their applicability in combinatorial molecular therapeutics [198, 199]. A number of reports have reinforced the notion that inhibition of a single transduction pathway may be insufficient since activation of alternative signaling cascades may conceal efficacy, and that it would be more advantageous to target integrated cancer signals, for example, VEGFR- and EGFR-interdependent pathways [170] and heparin-binding epidermal growth factor-like growth factor (HB-EGF) [200, 201]. Remarkably also, the mammalian target of rapamycin (mTOR) is a central intracellular kinase that not only orchestrates proliferation, survival, and angiogenic pathways, but has also been linked to resistance to EGFR antagonists, and thus mTOR inhibition could be explored to interfere with tumor growth and expansion at multiple levels [4, 83, 84, 92, 159, 170, 202–205]. Another multiple molecular targeting platform is provided by EGFR-induced EMT in EOC, possibly via mechanisms that incorporate estrogen signaling, E-cadherin downregulation and expression of matrix metalloproteinase-9 (MMP-9), and Snail transcription family members (SNAIL and SLUG) [79, 206, 207]. Additionally, oncolytic viruses engineered to deliver anti-EGFR antibodies to intraperitoneal ovarian cancer cells show great potential as a future gene therapeutic focus [208]. Irrespective of the prospects for molecular targeting of EGFR RTKs in ovarian cancer, resistance to EGFR inhibitors and unwanted adverse events in ovarian and non-ovarian tumors are major clinical concerns that need to be circumvented [16, 166, 174, 191, 209, 210].

### 3.4. Mucin 16 (MUC16) and Lewis X Mucin Determinant (OVX1)

The role of mucins in epithelial cancer, including ovarian cancer, pathogenesis is well established [211–213]. Mucin 16 (MUC16)—also called carcinoma antigen 125 (CA125)—is arguably the most consistently used biomarker for ovarian cancer [58, 59, 61, 64, 72, 73, 211, 214–221]. MUC16 is overexpressed in EOC and correlates with decreased E-cadherin, elevated N-cadherin and vimentin levels, and heightened invasivesness, tumorigenesis, tumor cell proliferation, and metastases, as confirmed by MUC16 knockdown which completely abolished the development of subcutaneous tumors in nude mice [222]. Interestingly, the C-terminal domain of MUC16 promotes cisplatin resistance and MUC16 selectively modulates the sensitivity of EOC cells to DNA-damaging drugs such as cyclophosphamide, doxorubicin and etoposide, effects validated by downregulation of cell surface MUC16 [223]. The strong interaction between MUC16 and mesothelin, a glycosylphosphatidylinositol- (GPI-) anchored glycoprotein, promotes cell adhesion and peritoneal metastasis of ovarian cancer cells [224, 225]. Furthermore, MUC16 suppresses natural killer (NK) cell-induced cytolysis in EOC patients, indicating that it compromises immune-mediated tumor surveillance and destruction [226]. In preclinical and clinical studies, antibodies and vaccines directed against mucins, evaluated for their potential to delay or limit the spread of tumor cells, produced significant survival benefits [211, 227–229]. The usefulness of MUC16 as a target antigen in ovarian carcinomas is hampered by cleavage and secretion of its extracellular domain. However, a recent study has shown that the introduction of a gene encoding a chimeric antigen receptor (CAR) targeted to the retained extracellular fraction (MUC-CD) and its retroviral transduction into human T cells specifically targets and lyses MUC-CD^+^ tumor cells and may thus signify an innovative design to adoptive immunotherapy of cancer [230–232]. In view of the previous assertions, MUC16 needs to be probed for its plausibility as a molecular target in the immunotherapy of ovarian cancers [233, 234].

MUC16 is used along with multiple serum biomarkers for the early detection and screening of ovarian cancer [235]. One such biomarker is the Lewis X mucin determinant (OVX1) which is increased in the majority of patients with EOC [59, 71, 72, 125, 218, 221, 236–238]. Monoclonal antibodies to OVX1 are internalized by ovarian cancer cell lines in vitro and may prove useful in the molecular targeting of this neoplasm with conjugated antibodies and immunotoxins [232, 238–241]. Curiously, alterations of the sugar moieties of the glycosylated Lewis X and Lewis Y antigens are frequent in epithelial ovarian cancers and, besides having obvious prognostic implications, may be prime arbiters along with extracellular matrix component interactions (e.g., *β*-integrin/fibronectin, CA125/mesothelin, CD44/hyaluronan) in CD44-mediated adhesion and peritoneal spreading (metastasis) of ovarian cancer cells [242]. These mechanisms should be explored as a molecular targeting principle in ovarian cancers.

### 3.5. The IL-6R-JAK-STAT3 Axis and Nuclear Factor Kappa-B (NF-*κ*B)

The upregulation of several proinflammatory cytokines in ovarian cancers confirms a link between inflammation and immunogenic-tumor microenvironment interactions in the increased risk of ovarian tumor initiation and progression [243–251]. IL-6 is a proinflammatory cytokine that modulates pleiotropic cellular and immune responses. Binding of the ligand, IL-6, to the *α*-subunit of its receptor (IL-6R) results in the formation of a heterodimeric complex (IL-6R/gp130) which activates Janus kinase (JAK) and various downstream effectors such as signal transducer and activator of transcription 3 (STAT3), SHP-2/Ras, mitogen-activated protein kinase (MAPK), and phosphatidylinositol-triphosphate kinase PI3K/Akt, critical for cell proliferation, apoptosis evasion and survival, drug resistance, and inactivation of tumor suppressors [252–258]. STAT3 is also activated by growth factor receptor signaling, including EGFR, HER2, VEGFR, PDGFR, IGFR, and FGFR [252]. Indeed, raised levels of IL-6 in ascites and serum from ovarian cancer patients correlate with cisplatin and paclitaxel resistance and poor disease prognosis [259], whereas blockade of STAT3 expression in ovarian cancer cells increases their sensitivity to paclitaxel [254]. The expression of IL-6 and its downstream signaling proteins is upregulated in ovarian clear cell adenocarcinoma (OCCA) and EOC [7, 260]. 

A recent study has shown unequivocally that siltuximab (a monoclonal anti-IL-6 antibody) significantly reduced ovarian cancer expression of STAT3 downstream proteins such as Mcl-1, Bcl-X(L), and survivin, implying proapoptotic effects. In the same study, metastatic and drug-resistant recurrent ovarian tumors expressed significantly higher IL-6 levels than primary ovarian cancer tissue [261]. By the same token, administration of sunitinib, a potent multikinase (VEGFR, PDGFR, and KIT) inhibitor, to two OCCA patients with progressive disease and refractory to conventional chemotherapy resulted in markedly lower levels of CA125 and notable reduction in tumor mass [7]. The possible mechanistic correlation for the favorable responses seen in these patients had been advanced as inhibition by sunitinib of IL-6, STAT3, and hypoxia-induced factor (HIF). Thus, the upregulation of the IL6-STAT3-HIF pathway in OCCA may be exploited as a biomarker to clinically differentiate OCCA from other ovarian tumor types [7], and inhibition of the IL-6-STAT3 signaling autocrine pathway may offer yet another molecular targeting strategy in the management of cisplatin- and paclitaxel-resistant ovarian cancers [259, 262]. The observation that crosstalk between the EGFR and IL-6R signaling through JAK/STAT3 mediates EMT in ovarian cancers further adds to the number of exploitable opportunities that are emerging to target the molecular intricacies that underscore the aggressive phenotype of ovarian cancer and its recurrence in patients [258, 263, 264]. Generic strategies to target the IL-6R-JAK-STAT3 signaling axis include receptor-ligand antagonists or antibodies, tyrosine or serine kinase inhibitors, transcription factor decoy (siRNA), physiological protein modulators of STAT3 activation, disrupters of STAT dimerization, inhibitors of STAT3 nuclear translocation, and target gene transcription [257].

Nuclear factor kappaB (NF-*κ*B) is a highly inducible transcription factor which regulates several inflammatory response and cancer signaling pathways [252, 265, 266]. NF-*κ*B is constitutively expressed in the majority of tumors, including ovarian cancer [80, 256, 257, 266]. Many cytokine-induced signaling pathways that control inflammation and cancer converge on NF-*κ*B and STAT3 [252]. The mammalian NF-*κ*B family comprises five members, namely, RelA (p65), RelB, cRel (Rel), NF-*κ*B1 (p50 and its precursor p105), and NF-*κ*B 2 (p52 and its precursor p100) which form homo- and heterodimers whose activities are regulated by two key NF-*κ*B activation pathways. In the first (classical or canonical) pathway, RelA:p50 dimers are sequestered in an inactive conformation in the cytoplasm through interactions with inhibitory proteins, I-*κ*B. Upon binding of ligands such as TNF-*α* or IL-1, viruses, genotoxic agents, and exposure to ionizing radiation, the I-*κ*B molecules become phosphorylated at specific serine residues by the I-*κ*B kinase complex (IKK, made up of two catalytic subunits, IKK*α* and IKK*β*, and a regulatory subunit, NEMO/IKK*γ*) which results in their ubiquitylation and proteasomal degradation. The liberated RelA:p50 dimers translocate to the nucleus to activate transcription of several target genes that regulate innate immunity and inflammation. In the second (alternative or non-canonical) pathway which is stimulated almost exclusively by members of the TNF superfamily, an upstream NF-*κ*B-inducing kinase (NIK) activates IKK*α*, causing phosphorylation and proteasomal processing of p100, the principal RelB inhibitor, followed by RelB:p52 and RelB:p50 nuclear translocation and binding to genes responsible for regulating development, organization, and function of secondary lymphoid organs, B-cell maturation, and survival. Even though many genes are regulated by STAT3 and NF-*κ*B, these two master regulators both favor the transcriptional activation of protumorigenic and antiapoptotic genes such as *Bcl-xL*, *Bcl-2,* and *c-IAP2*, while *A1* and *c-FLIP* genes are predominantly NF-*κ*B-dependent and *Mcl-1* and *survivin* genes are STAT3-dependent [252, 256, 265, 266]. NF-*κ*B (RelA/p65) is overexpressed in advanced-stage metastatic serous ovarian carcinoma, and its localization to the nucleus is associated with poor PFS [267]. Using specimens from patients with IKK*β*-positive ovarian tumors and ovarian cancer cell lines, a recent study showed that activation of the NF-*κ*B pathway by downregulating IKK*β* activity with highly specific kinase inhibitors or through short hairpin RNA (shRNA), depletion of IKK*β* correlated not only with a number of cellular expressions associated with the invasive phenotype of this cancer, but also with poor OS [80]. These findings are in agreement with the notion that constituent expression of NF-*κ*B in OCSCs, which may be the trigger of chemoresistance and disease recurrence, can be targeted by inactivation of NF-*κ*B signaling [25, 247]. 

Although IL-6 signaling has been studied extensively in ovarian cancers, several reports have indicated the involvement of many other interleukins in the development of this neoplasm [248, 252]. These will not be considered further in this review, except to mention that IL-8 has previously been identified to have autocrine growth factor, tumorigenic and angiogenic effects in human ovarian cancer [268–273], but conflicting reports have also appeared [274]. Particularly noteworthy is the fact that activation of G-protein-coupled receptor protease-activated receptor-1 (PAR1) by matrix metalloproteinase (MMP1) is a principal promoter of angiogenesis and metastasis in peritoneal mouse models of ovarian cancer. In ovarian carcinoma cells, activated MMP1-PAR1 induces the release of angiogenic factors such as interleukin-8 (IL-8) and growth-regulated oncogene-alpha (GRO-*α*) which, through paracrine signaling, act on endothelial CXCR1/2 to effect endothelial cell proliferation, tube formation, and migration [110]. This pathway may be targeted to identify novel ovarian cancer therapies.

### 3.6. PI3K/AKT/mTOR Cell Signaling Pathway

The mammalian target of rapamycin (mTOR) is a central intracellular kinase that coordinates mitogenic, angiogenic, antiapoptotic, and survival pathways in cancers through crosstalk with VEGF, HIF-1, and the EGFR/ErbB family of RTKs [202]. PI3K/Akt/mTOR signaling thus confers a selective survival advantage on tumor cells [397]. Activators of this pathway include defective tumor suppressor PTEN, upregulation or mutation of PI3K and AKT, and ligand binding to growth factor receptors. Mutation or amplification of PI3K or Akt triggers mTOR phosphorylation and increased ovarian tumor cell survival [398]. A recent study has shown that PI3K/AKT/mTOR signaling is involved in EOC development and resistance to cisplatin, since downregulation of AKT with triciribine or shRNA transfection of ovarian cancer cells decreased their resistance to cisplatin via mTOR/survivin signaling [92]. In advanced-stage ovarian cancer, the mTOR pathway is upregulated, and hence its blockade will enhance ovarian cancer cell sensitivity to antitumor drugs [204]. In patients with serous ovarian carcinoma undergoing cisplatin-taxane-based therapy, activation of VEGFR2/AKT/mTOR pathway was significantly correlated with raised ascites levels and decreased OS [205]. mTOR has been implicated in the resistance of various cancers to EGFR inhibitors [202] and mTOR pathway activation is a poor prognosticator of EOC [84]. Furthermore, treatment of highly metastatic ovarian tumor cells with bikunin (BIK) or upregulating *BIK* gene expression in these cells significantly attenuated PI3K/p85 gene expression, and decreased their urokinase-type plasminogen activator- (uPA-) dependent invasive potential in nude mice [292]. Therefore, the molecular targeting of multiple signaling pathways such as EGFR, VEGFR, HIF-1, and PI3K/PTEN/AKT/mTOR may improve responses in recurrent and resistant ovarian cancers [4, 83, 92, 203, 205, 399–403].

### 3.7. ATP-Binding Cassette (ABC) Drug Transporters

Despite the encouraging response rates of ovarian cancer patients to a combination regimen of carboplatin and paclitaxel, most will experience recurrence and/or relapse. Disease recurrence is mostly associated with the development of multidrug resistance (MDR) which is mediated by the overexpression of tumor ATP-binding cassette (ABC) drug transporters. In ovarian cancer cells, the* ABCB1 (MDR1)* gene encodes P-glycoprotein, which targets to the luminal surface and actively effluxes a wide array of anticancer drugs, including carboplatin and paclitaxel [404–406]. P-glycoprotein expression has been shown to be a predictor of unfavorable response (recurrence) and poor survival in uniformly treated and followed cohorts of advanced ovarian cancer patients [407–409]. Reversal of MDR in ovarian cancer cell lines is possible with siRNA knockout of *ABCB1 (MDR1)* and *ABCB4 (MDR3)* genes [410, 411], combination drug treatments [412, 413], chitosan/pshRNA plasmid nanoparticle targeting of *MDR1* genes [414], and perturbation of P-glycoprotein N-glycosylation [415]. The prognostic value of *ABCB1* gene polymorphisms in ovarian cancer patients is conflicting, for example, whereas a recent study found that ABCB1 G2677T/A and ABCB1 C3435T gene polymorphisms did not correlate with survival and prognosis in Caucasian women with ovarian cancer [416, 417], another study found such a relationship [418]. Analogous earlier reports concluded that although *MDR1* expression profiles may be closely related to histologic subtype of ovarian cancer, they were not accurate predictors of survival [419, 420]. Remarkably, elevated expression of MDR-1 in tumor tissue sampled after first cytoreductive surgery was associated with a higher risk of brain metastases in women with epithelial ovarian, fallopian tube, or peritoneal cancer [421]. Noteworthy also is the observation that chemoresistance induced by IL-6R signaling correlated with enhanced expression of MDR genes (*MDR1 and GSTpi*), antiapoptotic proteins (Bcl-2, Bcl-xL, and XIAP), and upregulation of Ras/MEK/ERK and PI3K/Akt signaling [259]. Undoubtedly, more research is required to unravel the complex expression of the MDR phenotype in ovarian cancers.

## 4. Candidate Ovarian Cancer Biomarkers as Molecular Targets

Candidate biomarker profiles and the molecular basis for their targeting in ovarian cancers are summarized in Table 1.

## 5. Conclusion

This aim of this review was to present a broad overview of how improved diagnostic and prognostic specificity and sensitivity of tumor biomarkers and signaling molecules can be translated into more efficacious molecularly targeted therapies that will prevent resistance, recurrence, and relapse in ovarian cancer patients. The different types of ovarian cancers variously express the major hallmarks of cancer such as genomic instability, gain of oncogenes, loss of tumor suppressors, immeasurable self-renewal potential, epithelial-to-mesenchymal transition, and reversed mutational capacities, autocrine signaling and self-sufficiency in growth factor requirements, host immune co-option, escape from immune surveillance and natural killer cell mediated oncolysis, apoptosis evasion, increased DNA repair mechanisms, sustained angiogenesis, invasion, and metastatic spread. The rapid increase in our understanding of the molecular processes that regulate cancer signatures in general has raised an equally strong desire to eradicate ovarian cancer before resistance, recurrence, and relapse can set in and claim more lives. It is becoming increasingly evident that traditional approaches to ovarian cancer management such as surgical debulking and carboplatin-paclitaxel chemotherapy will have to be complemented with molecularly targeted and personalized treatment approaches to impact positively on PFS and OS rates. The molecular therapeutic targeting paradigm and the concept of synthetic lethality as exemplified by BRCA1/2 mutations and PARP inhibition offer profound opportunities for ovarian cancer drug development and discovery. The targeting of multiple signaling pathways such as VEGFR, EGFR, IL-6R-JAK-STAT3/NF-*κ*B, PI3K/AKT/mTOR, and ABC drug transporters in ovarian cancer may be an auspicious start to favourable PFS and OS outcomes. The Wnt/*β*-catenin signaling pathway should not be overlooked since it has recently been implicated in regulating the immunoreactivity and chemosensitivity to anticancer drugs in ovarian cancer cells, which may be a useful prognostic indicator in patients with ovarian cancer [422]. The interaction between MUC16 and MES should be seen as an opportunity to block intra- and extraperitoneal metastasis of highly aggressive ovarian cancers and to develop effective antibodies and vaccines against this type of cancer which is a major contributor to the high mortality rate among women worldwide. Finally, candidate or emerging biomarkers, especially HDACi and miRNAs, and their molecular interactions with cancer signaling pathways should be translated into cross-spectrum and individualized therapies for the different histological subtypes of ovarian cancer.

## Figures and Tables

**Table 1:** Candidate biomarker profiles and the molecular basis for their targeting in ovarian cancers.

Biomarker^†^	Molecular basis for biomarker targeting in ovarian cancer	References
M-CSF	Hematopoietic cytokine that stimulates differentiation, activation, and proliferation of monocyte and macrophages; can also act as an autocrine or paracrine growth factor for some epithelial cancers; promotes vasculogenesis; modulates CSCs, and can thus be targeted in OCSCs to induce immune-mediated tumor cell lysis; a phase II trial with GM-CSF and recombinant interferon gamma 1b (rIFN-*γ*1b) in women with recurrent, platinum-sensitive ovarian, fallopian tube, and primary peritoneal cancer produced reasonable OS.	[14, 16, 33, 34, 59, 275]

HNF-1*β*	Overexpressed in ovarian clear cell adenocarcinoma (OCCC); reduction of HNF-1*β* expression by RNA interference induces apoptotic cell death in ovarian OCCC cells; HNF-1*β* is hypomethylated in OCCC and can thus be targeted in ovarian cancers.	[276–280]

HE4	A glycoprotein highly expressed in ovarian cancers that might have a role in ovarian carcinogenesis; HE4 expression is highest in endometrioid and serous ovarian cancer	[214, 281, 282]

OPN	A glycophosphoprotein cytokine secreted by activated T-lymphocytes, macrophages, and leukocytes at the inflammation site; higher levels occur in patients with ovarian cancer versus normal control; correlates significantly with tumor response to surgery, chemotherapy, and disease recurrence; implicated in tumorigenesis, tumor invasion, metastasis, and poor prognosis; binding of OPN as an ECM component to integrin and CD44 receptors in the tumor microenvioronment regulates signaling cascades associated with adhesion, migration, invasion, chemotaxis, and cell survival; alternative splicing of OPN leads to 3 isoforms, OPNa, OPNb, and OPNc; the latter possess ovarian protumorigenic properties mediated by PI3K/Akt signaling pathway which serves as a critical cancer molecular target.	[14, 111, 283–285]

MES	Binding of MUC16 to MES, a GPI-anchored glycoprotein, is thought to facilitate cell adhesion and peritoneal metastasis of ovarian tumors; this function can be exploited as a molecular targeting strategy, for example, anti-MES antibodies, to limit the metastatic spread of the tumor; MES is an attractive candidate for adenoviruses-mediated gene therapy of ovarian cancers; diffuse mesothelin expression is associated with prolonged survival in patients with high-grade ovarian serous carcinoma.	[224, 225, 286, 287]

HP-*α*	Glycoprotein synthesized in the liver, but also present in ascites and serum of ovarian cancer patients; proteomic profiling identified HP-*α* as a potential biomarker with high specificity for ovarian cancer; high levels of this acute phase protein correlate with poor prognosis, but attenuate with chemotherapy—this mechanism should be explored further.	[14, 71, 288–291]

BIK	This glycosylated protease suppresses ovarian tumor cell invasion and metastasis by downregulating PI3K and Ca^2+^-dependent TGF-*β* signaling pathways; plasma BIK is a strong prognostic indicator of ovarian cancer; a combination of BIK and paclitaxel significantly reduced tumor burden and ascites in a mouse model of ovarian cancer; BIK overexpression has been shown to suppress TNF-induced apoptosis in ovarian cancer cells; BIK also downregulates uPA/R and HBP gene expression in ovarian cancer cells; other target genes of BIK include transcriptional regulators, oncogenes/tumor suppressor genes, signaling molecules, growth/cell cycle, invasion/metastasis, cytokines, apoptosis, ion channels, and ECM proteins; the evidence cited here underlines the applicability of BIK in therapeutic strategies targeting the inhibition of peritoneal invasion and dissemination of ovarian cancer.	[14, 292–299]

FR*α*	This protein is an alternative folate transporter which may confer an increased DNA synthesis and growth advantage on tumor cells; ovarian cancer patients have elevated blood levels of this protein, identified as a diagnostic marker and molecular target in high-grade, high-stage serous tumors; the status of FR*α* apparently does not change in response to chemotherapy and has no effect on overall patient survival; however, farletuzumab, a humanized monoclonal antibody against FR*α*, demonstrated anticancer efficacy in patients with platinum-refractory/resistant EOC; FR*α* expression is preserved on metastatic foci and recurrent tumors, suggesting that novel folate-targeted therapies may have therapeutic potential for the majority of women with newly diagnosed or recurrent ovarian cancer.	[300–304]

TTR	This is a highly sensitive biomarker used in the screening of prostate, lung, colorectal, and ovarian (PLCO) cancers; was found to be downregulated in grade 3 ovarian tumors; and has been validated for its high specificity and sensitivity in early-stage ovarian cancer; further research on TTR is needed to explore its molecular targeting possibilities.	[58, 71, 305–307]

I*α*I	The expression of this protein is reportedly upregulated in ovarian cancer patients and it is used mainly to complement MUC16/CA125 in the screening for EOC; however, proteomic analysis showed its levels to be significantly reduced in the urine of patients with ovarian carcinoma.	[14, 125, 308]

CRP	Is one of a panel of plasma biomarkers used for the identification of women with ovarian cancer and to significantly increase diagnostic performance compared to MUC16/CA125 used singly; raised serum levels of CRP is associated with high levels of Il-6 and haptoglobin, considered as adverse prognostic factors in ovarian cancer; CRP are also a marker of high-grade inflammation in advanced-stage ovarian cancer and anemia in EOC (i.e., CRP correlates negatively with hemoglobin levels); high levels of prediagnostic CRP may indicate an inflammation stage that precedes ovarian cancer development and might denote increased risk.	[235, 291, 309–313]

PRSS	This channel-activating serine protease is overexpressed in EOC; it is localized to the apical surface of normal epithelial cells and suppresses cancer cell invasion in vitro; in various cancer cell lines, PRSS downregulates EGFR signaling by cleaving its extracellular domain and hence interferes with cell proliferation and tumor expansion; this property should be investigated as a molecular target.	[14, 71, 72, 314–316]

CLDNs	Large family of integral membrane proteins essential for tight junction formation and function; CLDN3 and CLDN4 expression levels are upregulated in EOCs of all subtypes and correlate with MMP-2 activity; CLDNs may promote ovarian cancer invasion and metastasis; CLDN upregulation in ovarian carcinoma effusions is associated with poor survival; cells that overexpress CLDN4 exhibit low DNA methylation and high histone H3 acetylation of the critical CLDN4 promoter region, while the converse is true for cells that do not overexpress it; CLDN4-expressing EOC cells secrete proangiogenic factors (e.g., IL-8) and downregulate genes of the angiostatic IFN pathway; CLDN5 overexpression is associated with aggressive behavior in serous ovarian adenocarcinoma; CLDNs are, therefore, suitable biomarkers for different types of ovarian cancer and promising molecular targets for ovarian cancer therapy.	[317–326]

APOA1	Is the protein component of HDL; the *APOA1* gene is upregulated in chemoresistant EOC and has an established role in tumorigenesis; algorithmic proteomic profiling of postdiagnostic/pretreatment sera of women with ovarian cancer revealed that the ApoA1 and TTR combination yield high specifity, but low sensitivity as tumor markers; further investigations into the mechanistic roles of APOA1 in ovarian tumorigenesis are crucial for its consideration as a molecular target in ovarian cancer.	[306, 327]

LPA	Generated by the action of the enzyme, lysophospholipase; LPA is the ligand for GPCRs (LPAR2 and LPAR3) which are upregulated during ovarian tumorigenesis; LPA is a bioactive lipid central to the initiation and progression of ovarian cancer; LPA is preferable to MUC16/CA125 as a biomarker for the diagnosis, but not the prognosis of EOC; in human EOC tissues obtained from patients, LPA-induced POSTN (an ECM constituent, see the following) expression in cancer-associated stromal fibroblasts correlates with poor survival and recurrence; remarkably, LPA also regulates IL-6 expression and STAT3 phosphorylation via the Gi/PI3K-Akt/NF-*κ*B pathway in ovarian cancer cells; LPA enhances growth and invasion of ovarian cancer cells and tumor angiogenesis; active RTK and EGFR signaling is required for LPA-mediated Gi-dependent cellular responses in ovarian cancer cells; LPA antibodies, LPA antagonists, and LPAR gene silencing may thus be useful molecular targeting strategies in ovarian cancer.	[2, 268, 328–339]

POSTN	POSTN is an ECM protein which normally functions as a homophilic adhesion molecule in bone formation; 5 isoforms have so far been identified; targeted comparative glycotranscriptome analyses of ovarian cancer and normal ovarian tissues have shown that POSTN and thrombospondin may be useful biomarkers for specific tumor-specific glycan changes in benign ovarian adenomas, borderline ovarian adenocarcinomas, as well as malignant ovarian adenocarcinomas; POSTN binds to numerous cell-surface receptors, predominantly integrins, and signals effectively via the PI3K/Akt and other pathways to promote cancer cell survival, EMT, invasion, metastasis, and angiogenesis; ovarian cancer cells actively secrete the protein; interaction of the ligand, POSTN, with integrins facilitates ovarian cancer cell motility; antibodies directed against POSTN have been shown to inhibit growth and metastasis of subcutaneous and ovarian tumors derived from a POSTN-expressing ovarian cancer cell line; thus, POSTN represents a novel molecular-targeted therapy for ovarian cancer.	[330, 340–345]

KLK	Largest family of flanking proteases in the human genome, comprising at least 15 members; KLKs are secreted serine proteases that stimulate or inhibit tumor progression; KLK5-11 levels are typically elevated in sera of ovarian cancer patients and regarded as predictors of poor disease prognosis; aberrant *KLK* gene expressions in different types of ovarian cancers may complicate generalizations; for example, high tumor KLK6 protein expression correlates with inferior patient outcome in ovarian cancer, while raised KLK8 is an independent marker of favorable prognosis in ovarian cancer, whereas KLK5 levels are low in serum of patients with benign ovarian tumors; elevated KLK5 antigen in serum and ascitic fluid of ovarian cancer patients is a prognostic factor for PFS; KLK5-specific antibodies have been detected in patients with benign masses, borderline tumors, and ovarian carcinomas compared with healthy controls; the presence of KLK5 antibodies suggests that KLK5 might represent a possible target for immune-based therapies; KLK6 exemplifies the altered glycosylation hallmark of ovarian cancer; KLK7 is associated with negative characteristics of ovarian cancer, but is not considered an independent prognosticator for the disease; a combined panel of KLK6, KLK13, and MUC16/CA125 affords improved sensitivity in the detection of early stage ovarian cancer than MUC16/CA125 alone; KLKs have recently been shown to be subject to posttranscriptional control by multiple miRNAs which can be exploited in the differential diagnosis of ovarian cancer and as a molecular targeting opportunity.	[60, 346–365]

AGR2	This is a mucinous metastasis-inducing protein detectable in the plasma of ovarian cancer patients; elevated AGR2 levels in ovarian cancer patients are associated with disease stages II and III in both serous and nonserous tumors; AGR2 is thought to promote cell proliferation and migration; it is currently being validated for its diagnostic and prognostic significance in ovarian cancers.	[67, 366–368]

HDACs	Posttranslational modification of histones by HATs results in acetylation of the histone structure which exposes chromatin of transcriptionally active genes; the acetylation status of histones governs access of transcription factors to DNA and determines levels of gene expression; HDACs catalyze the removal of acetyl groups from histone tails and thus suppress transcription; accordingly, homeostatic control of HATs and HDACs activities is essential for maintaining nuclear and genomic stability; HDACs also act on various other transcription factors such as p53, Rb, and E2F1; HDACs are often activated or mutated in human cancers; in ovarian tumors, type-specific overexpression and roles for these enzymes have been delineated; for example, HDAC1 promotes cell proliferation whereas HDAC3 induces cell migration by downregulating E-cadherin; HDACs have become critical drug targets for cancer therapy and HDACi shows tremendous promise in preclinical and clinical trials (www.clinicaltrials.gov); SAHA (vorinostat, Zolinza) has been approved by the FDA for treatment of cutaneous T-cell lymphoma; HDACi promotes cell cycle arrest by inducing CDK inhibitor p21 (WAF1/CIP1); moreover, HDACi has pleiotropic actions, including the upregulation of proapoptotic proteins of Bcl-2 family (Bim, Bmf, Bax, Bak, and Bik) and downregulation of antiapoptotic proteins of Bcl-2 family (Bcl-2, Bcl-XL, Bcl-w, Mcl-1) and XIAP and survivin which may be significant in apoptosis targeting approaches [369, 370]; HDACi, such as NaB, SAHA, and TSA, enhanced in vitro ovarian cancer cell killing with concomitant increased mRNA expression of MDR1 but decreased mRNA expression of MRP1 and MRP2; the novel hydroxamic acid-derived HDACi, MHY218, has been shown to be more potent than SAHA in suppressing ovarian tumor cell viability and transplanted tumor growth in an in vivo tumor carcinomatosis model; MHY218 also raised expression levels of the cell cycle inhibitor, p21WAF1/CIP1, induced apoptosis via caspase-3 activation, and increased release of cytochrome c and Bax/Bcl-2 ratio; previously, similar results have been reported for another novel HDACi, apicidin; in view of the above, it is clear that HDACi is an emerging molecular-targeted approach to the management of ovarian cancer, but prudent forethought should be given to specific targeting of different HDAC family members, for example, HDAC1 and HDAC2 coregulator complexes, and more especially since acetylated HDAC1 can transregulate HDAC2 through heterodimerization.	[327, 371–386]

miRNAs^‡^	MicroRNAs belong to a family of endogenous, small RNAs (~22 nucleotides); these noncoding, yet functional RNAs are key regulators of coding genes in the human genome; microarray analysis of altered expression of miRNAs provides useful information on the ontogeny and differentiation status of various cancers; genomic and epigenetic modifications are known to deregulate miRNA expression in human EOC; a recent study showed that several miRNAs (let-7e, miR-30c, miR-125b, miR-130a, and miR-335) were differentially expressed and upregulated in paclitaxel- and cisplatin-resistant ovarian cancer cell lines and concluded that the development of drug resistance in ovarian cancer may be linked to distinct miRNA fingerprints that could be used as biomarkers to monitor disease prognosis; deregulation of miRNA-27a may correlate with the development of drug resistance by regulating the expression of MDR1/P-glycoprotein targeting HIPK2 in ovarian cancer cells; deregulation of miR-214, miR-199a, miR-200a, and miR-100 has also been demonstrated to occur in ovarian cancers; miR-214 promotes cell survival and cisplatin resistance by targeting the PTEN/Akt pathway; lack of miRNA-31 expression has been linked to a defective p53 pathway in serous ovarian cancer patients, raising hopes that treatment with miRNA-31 may offer an efficacious strategy in the management of such patients; miRNA-125a is a negative regulator of EMT since it induces reversion of highly invasive ovarian cancer cells from a mesenchymal to an epithelial histotype; this finding represents a landmark in ovarian cancer therapeutics since overexpression of EGFR is coupled to EMT in ovarian cancer cells which correlates with poor prognosis; the expression of miRNA-200 family members in ovarian tumors obtained from patients correlated with raised levels of *β*-tubulin and poor PFS to paclitaxel-based treatment; some miRNAs have been identified as putative tumor suppressor genes in ovarian tumors; thus specific miRNA signatures may be exploited as biomarkers for progression and recurrence of advanced stage ovarian carcinoma patients, and as molecular targets in ovarian cancer.	[68, 387–396]

^†^Granulocyte/macrophage-colony stimulating factor (G/M-CSF); hepatocyte nuclear factor-1*β* (HNF-1*β*); human epididymis protein 4 (HE4); osteopontin (OPN); mesothelin (MES); haptoglobin-*α* (HP-*α*); Bikunin (BIK); phosphoinositide-3-kinase (PI3K); transforming growth factor-beta (TGF-*β*); tumor necrosis factor (TNF); urokinase plasminogen activator and its receptor (uPA/R); hyaluronan-binding protein (HBP); extracellular matrix (ECM); folate receptor alpha (FR*α*); transthyretin (TTR); inter-*α*-trypsin inhibitor (I*α*I); C-reactive protein (CRP); prostasin (PRSS); claudin/s (CLDN/s); matrix metalloproteinase-2 (MMP-2); interferon (IFN); apoliprotein A1 (APOA1); high-density lipoprotein (HDL); lysophosphatidic acid (LPA); G-protein coupled receptors (GPCRs); receptor tyrosine kinase (RTK); periostin (POSTN, also called osteoblast specific factor 2, OSF2); kallikrein/s (KLKs); human anterior gradient 2 (AGR2); histone acetyltransferase/s (HAT/s); histone deacetylase/s (HDAC/s); histone deacetylase inhibitors (HDACi); suberoylanilide hydroxamic acid (SAHA); sodium butyrate (NaB); trichostatin A (TSA); multidrug-resistant protein (MDR1, P-glycoprotein); multidrug resistance-associated proteins 1 and 2 (MRP1/2); microRNAs (miRNAs); extracellular matrix (ECM); homeodomain-interacting protein kinase-2 (HIPK2); glycosylphosphatidylinositol (GPI). All these biomarkers are used in various multimodal combinations in the screening/detection of ovarian cancer in high risk women.^‡^For more information, see (http://www.sanger.ac.uk/Software/Rfam/mirna/).
